# Supplementary material for: Pyruvate Dehydrogenase Kinase 1 inhibition mediated oxidative phosphorylation enhancement in cartilage promotes osteoarthritis progression
Source: BMC Musculoskelet Disord. 2023 Jul 20;24:597. doi: 10.1186/s12891-023-06585-6 (PMC10357736; doi:10.1186/s12891-023-06585-6)
Supplement: Supplementary file 1 — Additional file 1. [file 12891_2023_6585_MOESM1_ESM.zip › MF.Gsea.1653627316242/GOMF_OXIDOREDUCTASE_ACTIVITY_ACTING_ON_CH_OH_GROUP_OF_DONORS.html]

Details for gene set GOMF\_OXIDOREDUCTASE\_ACTIVITY\_ACTING\_ON\_CH\_OH\_GROUP\_OF\_DONORS[GSEA]

|  || Dataset | 55457\_collapsed\_to\_symbols.cls.cls #OA\_versus\_normal.cls.cls #OA\_versus\_normal\_repos |
| Phenotype | cls.cls#OA\_versus\_normal\_repos |
| Upregulated in class | OA |
| GeneSet | GOMF\_OXIDOREDUCTASE\_ACTIVITY\_ACTING\_ON\_CH\_OH\_GROUP\_OF\_DONORS |
| Enrichment Score (ES) | 0.24279152 |
| Normalized Enrichment Score (NES) | 1.1646315 |
| Nominal p-value | 0.14854111 |
| FDR q-value | 0.60151076 |
| FWER p-Value | 1.0 |
Table: GSEA Results Summary

  

Fig 1: Enrichment plot: GOMF\_OXIDOREDUCTASE\_ACTIVITY\_ACTING\_ON\_CH\_OH\_GROUP\_OF\_DONORS      
 Profile of the Running ES Score & Positions of GeneSet Members on the Rank Ordered List

  

| SYMBOL | TITLE | RANK IN GENE LIST | RANK METRIC SCORE | RUNNING ES | CORE ENRICHMENT || 1 | DHRS7 | dehydrogenase/reductase 7 [Source:HGNC Symbol;Acc:HGNC:21524] | 45 | 1.005 | 0.0277 | Yes |
| 2 | MDH1 | malate dehydrogenase 1 [Source:HGNC Symbol;Acc:HGNC:6970] | 271 | 0.772 | 0.0340 | Yes |
| 3 | GRHPR | glyoxylate and hydroxypyruvate reductase [Source:HGNC Symbol;Acc:HGNC:4570] | 363 | 0.720 | 0.0492 | Yes |
| 4 | DHRS7B | dehydrogenase/reductase 7B [Source:HGNC Symbol;Acc:HGNC:24547] | 489 | 0.662 | 0.0600 | Yes |
| 5 | HSD11B2 | hydroxysteroid 11-beta dehydrogenase 2 [Source:HGNC Symbol;Acc:HGNC:5209] | 539 | 0.645 | 0.0762 | Yes |
| 6 | CBR1 | carbonyl reductase 1 [Source:HGNC Symbol;Acc:HGNC:1548] | 598 | 0.624 | 0.0910 | Yes |
| 7 | CYP27A1 | cytochrome P450 family 27 subfamily A member 1 [Source:HGNC Symbol;Acc:HGNC:2605] | 611 | 0.619 | 0.1093 | Yes |
| 8 | MDH2 | malate dehydrogenase 2 [Source:HGNC Symbol;Acc:HGNC:6971] | 615 | 0.618 | 0.1282 | Yes |
| 9 | UEVLD | UEV and lactate/malate dehyrogenase domains [Source:HGNC Symbol;Acc:HGNC:30866] | 670 | 0.601 | 0.1426 | Yes |
| 10 | HADHB | hydroxyacyl-CoA dehydrogenase trifunctional multienzyme complex subunit beta [Source:HGNC Symbol;Acc:HGNC:4803] | 675 | 0.599 | 0.1609 | Yes |
| 11 | HSD17B8 | hydroxysteroid 17-beta dehydrogenase 8 [Source:HGNC Symbol;Acc:HGNC:3554] | 704 | 0.593 | 0.1771 | Yes |
| 12 | DHRS1 | dehydrogenase/reductase 1 [Source:HGNC Symbol;Acc:HGNC:16445] | 778 | 0.575 | 0.1892 | Yes |
| 13 | HSD17B12 | hydroxysteroid 17-beta dehydrogenase 12 [Source:HGNC Symbol;Acc:HGNC:18646] | 795 | 0.570 | 0.2057 | Yes |
| 14 | AKR1A1 | aldo-keto reductase family 1 member A1 [Source:HGNC Symbol;Acc:HGNC:380] | 808 | 0.567 | 0.2223 | Yes |
| 15 | CBR3 | carbonyl reductase 3 [Source:HGNC Symbol;Acc:HGNC:1549] | 936 | 0.531 | 0.2288 | Yes |
| 16 | IDH2 | isocitrate dehydrogenase (NADP(+)) 2 [Source:HGNC Symbol;Acc:HGNC:5383] | 967 | 0.525 | 0.2428 | Yes |
| 17 | ALDH3A2 | aldehyde dehydrogenase 3 family member A2 [Source:HGNC Symbol;Acc:HGNC:403] | 1263 | 0.465 | 0.2342 | No |
| 18 | HSD17B4 | hydroxysteroid 17-beta dehydrogenase 4 [Source:HGNC Symbol;Acc:HGNC:5213] | 1354 | 0.450 | 0.2411 | No |
| 19 | ME1 | malic enzyme 1 [Source:HGNC Symbol;Acc:HGNC:6983] | 1746 | 0.392 | 0.2227 | No |
| 20 | ME2 | malic enzyme 2 [Source:HGNC Symbol;Acc:HGNC:6984] | 1870 | 0.377 | 0.2248 | No |
| 21 | HPGD | 15-hydroxyprostaglandin dehydrogenase [Source:HGNC Symbol;Acc:HGNC:5154] | 1941 | 0.368 | 0.2307 | No |
| 22 | AKR1C3 | aldo-keto reductase family 1 member C3 [Source:HGNC Symbol;Acc:HGNC:386] | 1996 | 0.361 | 0.2377 | No |
| 23 | VKORC1 | vitamin K epoxide reductase complex subunit 1 [Source:HGNC Symbol;Acc:HGNC:23663] | 2306 | 0.326 | 0.2237 | No |
| 24 | CBR4 | carbonyl reductase 4 [Source:HGNC Symbol;Acc:HGNC:25891] | 2565 | 0.295 | 0.2126 | No |
| 25 | SPR | sepiapterin reductase [Source:HGNC Symbol;Acc:HGNC:11257] | 2602 | 0.290 | 0.2188 | No |
| 26 | AKR7A2 | aldo-keto reductase family 7 member A2 [Source:HGNC Symbol;Acc:HGNC:389] | 2690 | 0.280 | 0.2207 | No |
| 27 | DHRS4 | dehydrogenase/reductase 4 [Source:HGNC Symbol;Acc:HGNC:16985] | 2708 | 0.279 | 0.2280 | No |
| 28 | IMPDH2 | inosine monophosphate dehydrogenase 2 [Source:HGNC Symbol;Acc:HGNC:6053] | 2867 | 0.264 | 0.2239 | No |
| 29 | HSD17B6 | hydroxysteroid 17-beta dehydrogenase 6 [Source:HGNC Symbol;Acc:HGNC:23316] | 2965 | 0.254 | 0.2242 | No |
| 30 | HSD11B1 | hydroxysteroid 11-beta dehydrogenase 1 [Source:HGNC Symbol;Acc:HGNC:5208] | 3000 | 0.250 | 0.2293 | No |
| 31 | HMGCR | 3-hydroxy-3-methylglutaryl-CoA reductase [Source:HGNC Symbol;Acc:HGNC:5006] | 3047 | 0.245 | 0.2333 | No |
| 32 | AKR1B1 | aldo-keto reductase family 1 member B [Source:HGNC Symbol;Acc:HGNC:381] | 3138 | 0.236 | 0.2336 | No |
| 33 | CRYL1 | crystallin lambda 1 [Source:HGNC Symbol;Acc:HGNC:18246] | 3292 | 0.221 | 0.2285 | No |
| 34 | ABCC4 | ATP binding cassette subfamily C member 4 [Source:HGNC Symbol;Acc:HGNC:55] | 3344 | 0.217 | 0.2312 | No |
| 35 | HAO1 | hydroxyacid oxidase 1 [Source:HGNC Symbol;Acc:HGNC:4809] | 3493 | 0.203 | 0.2259 | No |
| 36 | KCNAB1 | potassium voltage-gated channel subfamily A regulatory beta subunit 1 [Source:HGNC Symbol;Acc:HGNC:6228] | 3640 | 0.191 | 0.2204 | No |
| 37 | HSD3B1 | "hydroxy-delta-5-steroid dehydrogenase, 3 beta- and steroid delta-isomerase 1 [Source:HGNC Symbol;Acc:HGNC:5217]" | 3695 | 0.186 | 0.2220 | No |
| 38 | RDH14 | retinol dehydrogenase 14 [Source:HGNC Symbol;Acc:HGNC:19979] | 3952 | 0.160 | 0.2069 | No |
| 39 | DHRS11 | dehydrogenase/reductase 11 [Source:HGNC Symbol;Acc:HGNC:28639] | 4008 | 0.155 | 0.2075 | No |
| 40 | HSD17B10 | hydroxysteroid 17-beta dehydrogenase 10 [Source:HGNC Symbol;Acc:HGNC:4800] | 4060 | 0.150 | 0.2081 | No |
| 41 | RDH11 | retinol dehydrogenase 11 [Source:HGNC Symbol;Acc:HGNC:17964] | 4102 | 0.146 | 0.2094 | No |
| 42 | HADHA | hydroxyacyl-CoA dehydrogenase trifunctional multienzyme complex subunit alpha [Source:HGNC Symbol;Acc:HGNC:4801] | 4163 | 0.141 | 0.2091 | No |
| 43 | SORD | sorbitol dehydrogenase [Source:HGNC Symbol;Acc:HGNC:11184] | 4231 | 0.134 | 0.2081 | No |
| 44 | BDH2 | 3-hydroxybutyrate dehydrogenase 2 [Source:HGNC Symbol;Acc:HGNC:32389] | 4247 | 0.133 | 0.2110 | No |
| 45 | HSD3B2 | "hydroxy-delta-5-steroid dehydrogenase, 3 beta- and steroid delta-isomerase 2 [Source:HGNC Symbol;Acc:HGNC:5218]" | 4424 | 0.119 | 0.2010 | No |
| 46 | IDH3G | isocitrate dehydrogenase (NAD(+)) 3 non-catalytic subunit gamma [Source:HGNC Symbol;Acc:HGNC:5386] | 4504 | 0.113 | 0.1983 | No |
| 47 | ME3 | malic enzyme 3 [Source:HGNC Symbol;Acc:HGNC:6985] | 4613 | 0.104 | 0.1931 | No |
| 48 | ADH5 | "alcohol dehydrogenase 5 (class III), chi polypeptide [Source:HGNC Symbol;Acc:HGNC:253]" | 4720 | 0.094 | 0.1877 | No |
| 49 | DHRS3 | dehydrogenase/reductase 3 [Source:HGNC Symbol;Acc:HGNC:17693] | 4770 | 0.089 | 0.1866 | No |
| 50 | ADH1C | "alcohol dehydrogenase 1C (class I), gamma polypeptide [Source:HGNC Symbol;Acc:HGNC:251]" | 4908 | 0.079 | 0.1784 | No |
| 51 | LDHAL6B | lactate dehydrogenase A like 6B [Source:HGNC Symbol;Acc:HGNC:21481] | 5040 | 0.070 | 0.1703 | No |
| 52 | CTBP2 | C-terminal binding protein 2 [Source:HGNC Symbol;Acc:HGNC:2495] | 5066 | 0.067 | 0.1704 | No |
| 53 | HADH | hydroxyacyl-CoA dehydrogenase [Source:HGNC Symbol;Acc:HGNC:4799] | 5088 | 0.065 | 0.1708 | No |
| 54 | AKR7A3 | aldo-keto reductase family 7 member A3 [Source:HGNC Symbol;Acc:HGNC:390] | 5192 | 0.057 | 0.1645 | No |
| 55 | GPD1L | glycerol-3-phosphate dehydrogenase 1 like [Source:HGNC Symbol;Acc:HGNC:28956] | 5381 | 0.041 | 0.1511 | No |
| 56 | HSD17B11 | hydroxysteroid 17-beta dehydrogenase 11 [Source:HGNC Symbol;Acc:HGNC:22960] | 5776 | 0.010 | 0.1206 | No |
| 57 | AKR1D1 | aldo-keto reductase family 1 member D1 [Source:HGNC Symbol;Acc:HGNC:388] | 5836 | 0.006 | 0.1162 | No |
| 58 | AKR1C4 | aldo-keto reductase family 1 member C4 [Source:HGNC Symbol;Acc:HGNC:387] | 5843 | 0.005 | 0.1159 | No |
| 59 | RDH8 | retinol dehydrogenase 8 [Source:HGNC Symbol;Acc:HGNC:14423] | 5947 | -0.001 | 0.1079 | No |
| 60 | RDH5 | retinol dehydrogenase 5 [Source:HGNC Symbol;Acc:HGNC:9940] | 6277 | -0.026 | 0.0830 | No |
| 61 | ALDH3A1 | aldehyde dehydrogenase 3 family member A1 [Source:HGNC Symbol;Acc:HGNC:405] | 6413 | -0.036 | 0.0736 | No |
| 62 | G6PD | glucose-6-phosphate dehydrogenase [Source:HGNC Symbol;Acc:HGNC:4057] | 7082 | -0.086 | 0.0240 | No |
| 63 | IDH1 | isocitrate dehydrogenase (NADP(+)) 1 [Source:HGNC Symbol;Acc:HGNC:5382] | 7109 | -0.089 | 0.0247 | No |
| 64 | KCNAB3 | potassium voltage-gated channel subfamily A regulatory beta subunit 3 [Source:HGNC Symbol;Acc:HGNC:6230] | 7142 | -0.092 | 0.0251 | No |
| 65 | GPD2 | glycerol-3-phosphate dehydrogenase 2 [Source:HGNC Symbol;Acc:HGNC:4456] | 7206 | -0.097 | 0.0232 | No |
| 66 | GFUS | GDP-L-fucose synthase [Source:HGNC Symbol;Acc:HGNC:12390] | 7317 | -0.103 | 0.0178 | No |
| 67 | GPD1 | glycerol-3-phosphate dehydrogenase 1 [Source:HGNC Symbol;Acc:HGNC:4455] | 7420 | -0.112 | 0.0133 | No |
| 68 | DHRS9 | dehydrogenase/reductase 9 [Source:HGNC Symbol;Acc:HGNC:16888] | 7492 | -0.118 | 0.0114 | No |
| 69 | DCXR | dicarbonyl and L-xylulose reductase [Source:HGNC Symbol;Acc:HGNC:18985] | 7549 | -0.123 | 0.0108 | No |
| 70 | LDHC | lactate dehydrogenase C [Source:HGNC Symbol;Acc:HGNC:6544] | 7603 | -0.127 | 0.0106 | No |
| 71 | LDHB | lactate dehydrogenase B [Source:HGNC Symbol;Acc:HGNC:6541] | 7718 | -0.135 | 0.0059 | No |
| 72 | HSD17B1 | hydroxysteroid 17-beta dehydrogenase 1 [Source:HGNC Symbol;Acc:HGNC:5210] | 7940 | -0.150 | -0.0067 | No |
| 73 | EHHADH | enoyl-CoA hydratase and 3-hydroxyacyl CoA dehydrogenase [Source:HGNC Symbol;Acc:HGNC:3247] | 7968 | -0.153 | -0.0041 | No |
| 74 | AKR1C1 | aldo-keto reductase family 1 member C1 [Source:HGNC Symbol;Acc:HGNC:384] | 8198 | -0.171 | -0.0167 | No |
| 75 | H6PD | hexose-6-phosphate dehydrogenase/glucose 1-dehydrogenase [Source:HGNC Symbol;Acc:HGNC:4795] | 8303 | -0.178 | -0.0193 | No |
| 76 | AKR1B10 | aldo-keto reductase family 1 member B10 [Source:HGNC Symbol;Acc:HGNC:382] | 8420 | -0.187 | -0.0226 | No |
| 77 | HSD17B14 | hydroxysteroid 17-beta dehydrogenase 14 [Source:HGNC Symbol;Acc:HGNC:23238] | 8441 | -0.189 | -0.0182 | No |
| 78 | ADH7 | "alcohol dehydrogenase 7 (class IV), mu or sigma polypeptide [Source:HGNC Symbol;Acc:HGNC:256]" | 8906 | -0.225 | -0.0475 | No |
| 79 | ADH1B | "alcohol dehydrogenase 1B (class I), beta polypeptide [Source:HGNC Symbol;Acc:HGNC:250]" | 9036 | -0.235 | -0.0503 | No |
| 80 | LIPF | "lipase F, gastric type [Source:HGNC Symbol;Acc:HGNC:6622]" | 9074 | -0.238 | -0.0459 | No |
| 81 | CYP4F3 | cytochrome P450 family 4 subfamily F member 3 [Source:HGNC Symbol;Acc:HGNC:2646] | 9224 | -0.250 | -0.0497 | No |
| 82 | PHGDH | phosphoglycerate dehydrogenase [Source:HGNC Symbol;Acc:HGNC:8923] | 9245 | -0.252 | -0.0435 | No |
| 83 | ADH1A | "alcohol dehydrogenase 1A (class I), alpha polypeptide [Source:HGNC Symbol;Acc:HGNC:249]" | 9283 | -0.254 | -0.0385 | No |
| 84 | DHRS2 | dehydrogenase/reductase 2 [Source:HGNC Symbol;Acc:HGNC:18349] | 9363 | -0.261 | -0.0366 | No |
| 85 | ADH6 | alcohol dehydrogenase 6 (class V) [Source:HGNC Symbol;Acc:HGNC:255] | 9504 | -0.274 | -0.0390 | No |
| 86 | CYP4F2 | cytochrome P450 family 4 subfamily F member 2 [Source:HGNC Symbol;Acc:HGNC:2645] | 9599 | -0.283 | -0.0376 | No |
| 87 | RDH16 | retinol dehydrogenase 16 [Source:HGNC Symbol;Acc:HGNC:29674] | 9900 | -0.312 | -0.0514 | No |
| 88 | BDH1 | 3-hydroxybutyrate dehydrogenase 1 [Source:HGNC Symbol;Acc:HGNC:1027] | 10049 | -0.326 | -0.0528 | No |
| 89 | SRD5A2 | steroid 5 alpha-reductase 2 [Source:HGNC Symbol;Acc:HGNC:11285] | 10149 | -0.336 | -0.0501 | No |
| 90 | CTBP1 | C-terminal binding protein 1 [Source:HGNC Symbol;Acc:HGNC:2494] | 10188 | -0.339 | -0.0426 | No |
| 91 | UGDH | UDP-glucose 6-dehydrogenase [Source:HGNC Symbol;Acc:HGNC:12525] | 10279 | -0.349 | -0.0388 | No |
| 92 | KCNAB2 | potassium voltage-gated channel subfamily A regulatory beta subunit 2 [Source:HGNC Symbol;Acc:HGNC:6229] | 10387 | -0.360 | -0.0360 | No |
| 93 | IDH3A | isocitrate dehydrogenase (NAD(+)) 3 catalytic subunit alpha [Source:HGNC Symbol;Acc:HGNC:5384] | 10426 | -0.364 | -0.0277 | No |
| 94 | GCLM | glutamate-cysteine ligase modifier subunit [Source:HGNC Symbol;Acc:HGNC:4312] | 10439 | -0.366 | -0.0173 | No |
| 95 | GLYR1 | glyoxylate reductase 1 homolog [Source:HGNC Symbol;Acc:HGNC:24434] | 10807 | -0.406 | -0.0333 | No |
| 96 | PGD | phosphogluconate dehydrogenase [Source:HGNC Symbol;Acc:HGNC:8891] | 10845 | -0.410 | -0.0235 | No |
| 97 | IMPDH1 | inosine monophosphate dehydrogenase 1 [Source:HGNC Symbol;Acc:HGNC:6052] | 11023 | -0.433 | -0.0239 | No |
| 98 | LDHA | lactate dehydrogenase A [Source:HGNC Symbol;Acc:HGNC:6535] | 11134 | -0.448 | -0.0186 | No |
| 99 | HSD17B2 | hydroxysteroid 17-beta dehydrogenase 2 [Source:HGNC Symbol;Acc:HGNC:5211] | 11148 | -0.451 | -0.0057 | No |
| 100 | IDH3B | isocitrate dehydrogenase (NAD(+)) 3 non-catalytic subunit beta [Source:HGNC Symbol;Acc:HGNC:5385] | 11304 | -0.472 | -0.0032 | No |
| 101 | KDSR | 3-ketodihydrosphingosine reductase [Source:HGNC Symbol;Acc:HGNC:4021] | 11473 | -0.498 | -0.0008 | No |
| 102 | NSDHL | NAD(P) dependent steroid dehydrogenase-like [Source:HGNC Symbol;Acc:HGNC:13398] | 11670 | -0.527 | 0.0002 | No |
| 103 | HSD17B7 | hydroxysteroid 17-beta dehydrogenase 7 [Source:HGNC Symbol;Acc:HGNC:5215] | 11723 | -0.537 | 0.0128 | No |
| 104 | HAO2 | hydroxyacid oxidase 2 [Source:HGNC Symbol;Acc:HGNC:4810] | 11987 | -0.591 | 0.0106 | No |
| 105 | FASN | fatty acid synthase [Source:HGNC Symbol;Acc:HGNC:3594] | 12194 | -0.643 | 0.0144 | No |
| 106 | L2HGDH | L-2-hydroxyglutarate dehydrogenase [Source:HGNC Symbol;Acc:HGNC:20499] | 12208 | -0.648 | 0.0335 | No |
| 107 | HSD17B3 | hydroxysteroid 17-beta dehydrogenase 3 [Source:HGNC Symbol;Acc:HGNC:5212] | 12253 | -0.662 | 0.0506 | No |
Table: GSEA details [plain text format]

  

Fig 2: GOMF\_OXIDOREDUCTASE\_ACTIVITY\_ACTING\_ON\_CH\_OH\_GROUP\_OF\_DONORS      
 Blue-Pink O' Gram in the Space of the Analyzed GeneSet

  

Fig 3: GOMF\_OXIDOREDUCTASE\_ACTIVITY\_ACTING\_ON\_CH\_OH\_GROUP\_OF\_DONORS: Random ES distribution      
 Gene set null distribution of ES for **GOMF\_OXIDOREDUCTASE\_ACTIVITY\_ACTING\_ON\_CH\_OH\_GROUP\_OF\_DONORS**
